# Foodborne Transmission of Nipah Virus, Bangladesh

**DOI:** 10.3201/eid1212.060732

**Published:** 2006-12

**Authors:** Stephen P. Luby, Mahmudur Rahman, M. Jahangir Hossain, Lauren S. Blum, M. Mushtaq Husain, Emily Gurley, Rasheda Khan, Be-Nazir Ahmed, Shafiqur Rahman, Nazmun Nahar, Eben Kenah, James A. Comer, Thomas G. Ksiazek

**Affiliations:** * International Centre for Diarrheal Disease Research, Dhaka, Bangladesh;; † Institute for Epidemiology Disease Control and Research, Dhaka, Bangladesh;; ‡ Centers for Disease Control and Prevention, Atlanta, Georgia, USA

**Keywords:** Nipah Virus, Bangladesh, Encephalitis, Chiroptera, Epidemiology, Disease Outbreaks, Research

## Abstract

We investigated an outbreak of encephalitis in Tangail District, Bangladesh. We defined case-patients as persons from the outbreak area in whom fever developed with new onset of seizures or altered mental status from December 15, 2004, through January 31, 2005. Twelve persons met the definition; 11 (92%) died. Serum specimens were available from 3; 2 had immunoglobulin M antibodies against Nipah virus by capture enzyme immunoassay. We enrolled 11 case-patients and 33 neighborhood controls in a case-control study. The only exposure significantly associated with illness was drinking raw date palm sap (64% among case-patients vs. 18% among controls, odds ratio [OR] 7.9, p = 0.01). Fruit bats (*Pteropus giganteus*) are a nuisance to date palm sap collectors because the bats drink from the clay pots used to collect the sap at night. This investigation suggests that Nipah virus was transmitted from *P. giganteus* to persons through drinking fresh date palm sap.

## Abstract 2

TOC summary line: Nipah virus was likely transmitted from fruit bats to humans by drinking fresh date palm sap.

Nipah virus was first recognized in a large human outbreak that affected 283 persons and caused 109 deaths in Malaysia in 1999 (*1**,**2*). The outbreak was preceded by a large Nipah outbreak among pigs (*3*). Contact with sick pigs was the primary risk factor for human Nipah virus infection (*4*). The porcine outbreak, in turn, was thought to be caused by transmission of Nipah virus from fruit bats to pigs. Antibodies against Nipah virus were identified in the 2 native Pteropus species (*5*), and the virus was subsequently isolated from pooled urine samples from a P. hypomelanus colony on Tioman Island, Malaysia (*6*). The most likely initiating event was that a fruit bat that was shedding Nipah virus in its saliva dropped a piece of partially eaten fruit into a pig sty, and 1 or more of the pigs became infected (*1**,**7*). Genetic characterization of the Nipah virus strains isolated from pigs in the Malaysia outbreak identified 2 strains, 1 of which was identified in humans, and 1 of which may have given rise to the other through genetic drift (*8*). These findings suggest that as few as 1 or 2 instances of spillover of Nipah virus from bats to pigs occurred. No further cases of Nipah virus have been reported in Malaysia since 1999.

Four outbreaks of Nipah virus have been recognized in central and west Bangladesh from 2001 through 2004 (*9**–**11*) (Figure 1). Each outbreak occurred between January and May. Different outbreaks have been associated with different exposures. In the first outbreak in Meherpur in 2001, Nipah case-patients were significantly more likely to have had contact with a sick cow and contact with the secretions of an ill person than were controls (*9*). In Naogaon in 2003, case-patients were more likely than controls to have had contact with a herd of pigs that had passed through the area before the outbreak (*12*). In the 2004 outbreak in Goalando, Nipah case-patients were significantly more likely to have climbed trees and to have had contact with ill persons than were controls (*13*). In the 2004 outbreak in Faridpur, contact with ill persons was the primary risk for human Nipah disease (*10*).

**Figure 1:**
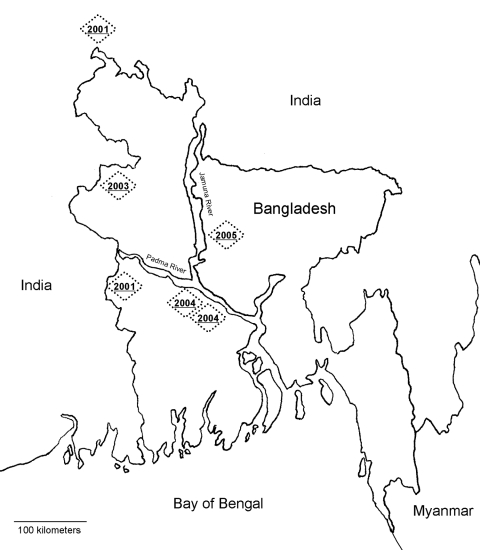
Location and dates of confirmed Nipah outbreaks in and near Bangladesh.

Substantial data implicate flying foxes (Pteropus spp.) as the natural reservoir of Nipah virus. Investigations of Pteropus spp. in Malaysia, Cambodia, and Thailand have consistently identified antibodies against Nipah virus (*5**,**14**–**16*). It has been isolated from Pteropus spp. bats in Malaysia, Cambodia, and Thailand (*6**,**15**,**16*). P. giganteus is the only Pteropus species present in Bangladesh. In the Naogaon investigation, 2 of 19 P. giganteus specimens had antibody against Nipah virus. None of 31 other animals from various species had Nipah antibodies (*9*). Strains of Nipah virus isolated from affected persons in Bangladesh have substantial genetic diversity (*17*). The repeated outbreaks in Bangladesh and the genetic diversity of Nipah virus isolated from affected persons in Bangladesh suggest substantial diversity of the virus in the wildlife reservoir and repeated spillover of the virus from its reservoir to the human population.

On January 11, 2005, government health workers in Tangail District reported that 8 previously healthy persons from Basail Upazila (subdistrict) had died within the preceding week from an illness characterized by fever and mental status changes. The Institute for Epidemiology Disease Control and Research (IEDCR) of the government of Bangladesh immediately launched an investigation and 5 days later invited the International Centre for Diarrheal Disease Research, Bangladesh (ICDDRB) to assist. The objectives of the investigation were to determine the cause of the outbreak, identify risk factors for development of illness, and develop strategies for prevention.

## Methods

### Case Identification

From January 11 onward, government health workers at the Basail Health Center recorded the names and basic clinical information and collected a blood sample from all patients who sought treatment for fever, seizures, or mental status changes. They followed up each ill person at least once per week until he or she recovered. Government health authorities and hospital medical directors in surrounding areas were notified of the outbreak and encouraged to contact the IEDCR if any patients with symptoms of encephalitis from Tangail District sought treatment in a healthcare institution outside of the district. Basail Upazila is composed of 6 unions. Ultimately, the study team defined a case-patient with outbreak-associated encephalitis as a person who lived or traveled in Habla Union, Basail Upazila, Tangail District, Bangladesh, in whom a fever developed and who had new onset of seizures or altered mental status between December 15, 2004, and January 31, 2005.

### In-Depth Interviews

An experienced anthropology team conducted in-depth interviews with the families of each case-patient. The objectives were 1) to explore potentially relevant exposures; 2) to assist in framing questions for the case-control questionnaire within the context of the activities, understanding, and language of local residents; and 3) to identify appropriate proxy respondents for each case-patient. Topics covered in the interviews included details on exposure to ill or dead persons; purchase and consumption of date palm sap; contact with animals, especially sick animals; availability and consumption of locally grown fruits, vegetables, and flowering plants; and presence and behavior of fruit bats in the area. The anthropology team interviewed collectors of date palm sap in detail about the context and process of date palm sap collection, preparation of the sap for consumption, and sales and distribution of the sap.

### Case-Control Study

On the basis of the findings from the in-depth study, a closed-ended questionnaire was developed and translated into Bengali. Five interviewers with extensive prior experience in administering close-ended questionnaires were trained in a 2-day course on a standardized method to request informed consent and administer the questionnaire. The interviewers then pretested the questionnaire in the presence of supervisors on a sample population from a nearby village that was not included in the study. After final revisions, the interviewers administered the questionnaire to each case-patient or his or her proxy(ies). Controls were identified by visiting the next closest house to the case-patient, confirming that no one in the house met the case definition, identifying the household resident closest in age to the case-patient, and then seeking consent to administer the questionnaire. Only 1 control was enrolled per household. If the household resident closest in age to the case-patient declined to participate in the study, no other person in the household was sought as a control. This process was repeated at the next closest household until 3 controls were enrolled for each case-patient.

Proxy respondents were identified for each case-patient who had died or was unable to respond. Proxy respondents included spouses, family members, and friends. Multiple proxy respondents were common; for example, a neighborhood playmate could be aware of food exposures that a parent might not be.

### Mapping

We measured the location of key features in the outbreak by using global positioning system sensing. We then superimposed these locations on publicly available government maps.

### Laboratory Methods

Whole blood specimens were transported on wet ice to the laboratory at ICDDRB, where they were centrifuged; the separated serum was stored at –70°C. Serum samples were shipped on dry ice to the Centers for Disease Control and Prevention (CDC) and tested with an immunoglobulin M (IgM) capture enzyme immunoassay (EIA) that detects Nipah IgM antibodies and an indirect EIA for Nipah IgG antibodies (*18*). Nipah (Malaysia prototype) virus antigen was used in both assays.

### Statistics

We used odds ratios to estimate the association of each exposure with disease. To assess whether observations departed from what would be expected by chance, we used the χ^2^ test when expected cell sizes were >5 and the Fisher exact test when expected cell sizes were <5. We calculated exact mid-p 95% confidence limits around the odds ratio. We used an unmatched analysis because neighbors were chosen as controls to ensure that controls arose from the same population as case-patients and not to control for confounding factors. Indeed, all case-patients and controls lived within the same area. We enrolled persons closest in age, not to control confounding by age but rather to provide simple guidelines to the interviewers that would prevent the common tendency to disproportionately enroll heads of households. Only 1 exposure was significantly associated with illness in the initial analysis. To account for the lack of independence among the exposures of the 3 case-patients that occurred in the same household, we used a generalized estimating equations model with an exchangeable correlation matrix (*19*).

### Ethics

The investigation team developed messages based on evidence from prior outbreaks on what steps family members could take to prevent person-to-person transmission of Nipah virus. During the outbreak, government health workers actively disseminated these messages. In addition, at the end of each in-depth interview carried out by the anthropology team, messages on steps to prevent person-to-person transmission of Nipah virus were directly communicated to case-patient families. Informed consent was requested of all study participants or their proxies. Because an emergency outbreak investigation was being conducted, the protocol was not reviewed by a human subjects committee.

## Results

Government health workers identified 124 persons within the outbreak area in whom fever developed during January 2005. Of these, 12 persons met the outbreak-associated encephalitis case definition. Among case-patients, the most common accompanying symptom was lack of consciousness (Table 1). The patients' median age was 16 years (range 5–85 years); 7 (58%) were male. Eleven (92%) of the persons who met the case definition died. Death occurred a median of 5 days (range 4–9 days) after the first symptom of illness was reported.

**Table 1:** Symptoms and signs of persons with outbreak-associated encephalitis, Habla Union, Bangladesh, January 2005

Symptom and signs	No. (%)
Fever	12 (100)
Death	11 (92)
Lack of consciousness	9 (75)
Headache	5 (42)
Vomiting	5 (42)
Seizures	4 (33)
Difficulty breathing	1 (8)

**Figure 2:**
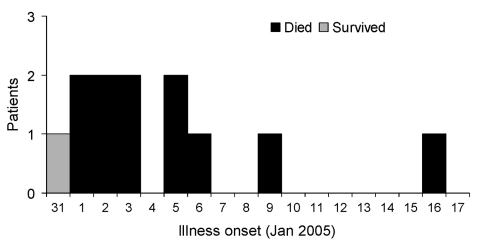
Dates of Illness onset, encephalitis outbreak, Habla Union, Bangladesh.

**Figure 3:**
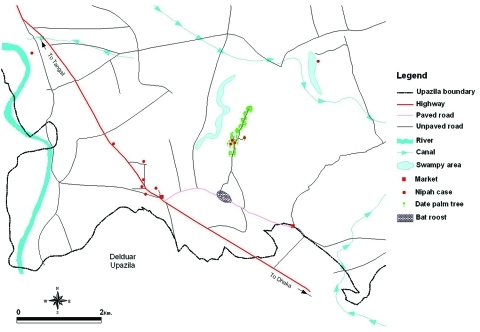
Outbreak area, Habla Union, Basail Upazila.

The onset of illness for all of the case-patients occurred within 17 days, and all but the last case occurred after 10 days (Figure 2). All of the case-patients lived within 8 km of each other (Figure 3). Three cases occurred in a single household.

Serum specimens were collected from 3 persons who met the outbreak-associated encephalitis case definition (Table 2). Two case-patients had IgM antibodies against Nipah virus by capture EIA. These 2 case-patients had blood collected 8 and 17 days after illness onset. The patient without detectable IgM antibody had his blood collected 2 days after illness onset. Both patients with IgM antibody also had Nipah IgG antibodies detected. Serum was also collected from 20 residents of the outbreak-affected community who had fever but did not meet the outbreak-associated encephalitis case definition. All 20 of these specimens were negative for Nipah IgM and IgG antibodies.

**Table 2:** Serum Nipah capture antibody results, encephalitis outbreak, Habla Union, Bangladesh*

Age, y	Illness onset	Date of serum collection	Outcome	Nipah IgM	Nipah IgG
6	Dec 31, 2004	Jan 17, 2005	Survived	+	+
25	Jan 9, 2005	Jan 11, 2005	Died	–	–
12	Jan 16, 2005	Jan 24, 2005	Died	+	+

*Ig, immunoglobulin.

### Case-Control Study

Interviewers enrolled 11 patients who met the outbreak-associated encephalitis case definition and 33 matched controls. One patient was excluded because we could not identify proxy respondents with thorough knowledge of his exposures. Proxy respondents were used for all case-patient interviews and for 6 (17%) control interviews.

The only exposure that was significantly associated with illness was drinking raw date palm sap (64% among cases versus 18% among controls, odds ratio [OR] 7.9, p = 0.01, Table 3). Of the 13 persons who reported consuming date palm sap, 11 knew the location where the sap had been harvested. Ten (91%) reported that the sap was harvested from a single village. None of the study participants were harvesters of date palm sap; none reported drinking the date palm sap directly from the collection container.

**Table 3:** Bivariate analysis of risk factors for encephalitis Habla Union, Tangail District, Bangladesh, 2005

Risk factor	Case-patients with this risk factor, n = 11; no. (%)	Controls with this risk factor, n = 33; no. (%)	Odds ratio	95% Confidence limits*	p value†
Male sex	6 (55)	16 (49)	1.3	0.31– 5.4	0.73
Climbed trees	3 (27)	11(33)	0.8	0.14–3.4	1.0
Physical contact with living animal					
Pig	0	0	Undefined	1.0	
Fruit bat	0	0	Undefined	1.0	
Cow	5 (46)	21 (64)	0.48	0.11–2.0	0.31
Goat	2 (18)	6 (18)	1.00	0.12–5.8	1.0
Sheep	0	2 (6)	0	0, 11	1.0
Chicken	5 (46)	9 (27)	2.2	0.50–9.4	0.29
Duck	3 (27)	7 (21)	1.4	0.24–6.7	0.69
Cat	1 (9)	10 (30)	0.23	0.01–1.7	0.24
Physical contact with any sick animal	4 (36)	4 (12)	4.1	0.7–22	0.09
Physical contact with sick chicken	2 (18)	2 (6)	3.4	0.3–36	0.26
Killed a sick animal	0	1 (3)	0	0–57	1.0
Ate an animal that had been sick at the time it was killed	0	1 (3)	0	0–57	1.0
Drank raw date palm sap	7 (64)	6 (18)	7.9	1.6–38	0.01
Ate					
Banana	3 (27)	11 (33)	0.75	0.14–3.4	1.00
Papaya	1 (9)	7 (21)	0.37	0.01–2.9	0.66
Starfruit	2 (18)	8 (24)	0.7	0.09–3.8	1.0
Guava	5 (46)	14 (42)	1.1	0.27–4.6	1.0
Tamarind	1 (9)	3 (9)	1.0	0.03–11	1.0
Buroy	2 (18)	6 (18)	1.0	0.12–5.8	1.00
Traveled outside subdistrict	4 (36)	10 (30)	1.3	0.28–5.6	0.73
Touched someone with fever and altered mental status who later died	0	7 (21)	0.0	0.0–2.0	0.16
Been in the same room with someone with fever and altered mental status who later died	2 (18)	9 (27)	0.59	0.08–3.2	0.70

*Exact mid-p. †All p values are 2-tailed. Fisher exact test used when expected cell size <5.

A greater proportion of case-patients than controls reported physical contact with sick animals, although this difference may have been due to chance (36% vs. 12%, OR 4.1, p = 0.09). Two case-patients had contact with a sick hen, 1 with a sick cat, and 1 with a sick sheep. None of the 44 case-patients or controls reported physical contact with pigs or fruit bats, and none had eaten bats. Case-patients were no more likely than controls to have climbed trees or to have had contact with ill persons who later died. In the general estimated equation model that adjusted for the 3 cases clustered in the same household, drinking raw date palm sap was significantly associated with illness (adjusted OR 5.6, 95% confidence limits 1.7–7.9, p = 0.03).

### Qualitative Findings

Date palm sap collectors explained that harvesting is a seasonal occupation that, in this region, begins in mid-December with the first cold night and continues through mid-February. At the beginning of the season, the bark is shaved off on 1 side of the tree (Phoenix sylvestris) near the top in a V shape, and a small hollow bamboo tap is placed at the base of the V. In the late afternoon, date palm sap collectors climb the tree, scrape the area where the bark is denuded so the sap can flow freely, and tie a 2- to 4-L clay pot under the tap. During the night, as the palm sap rises to the top of the tree, some leaks out where the bark is denuded, flows through the tap, and drips into the clay pot. Palm sap collectors climb the trees between 5:00 a.m. and 6:00 a.m. to gather the clay pots.

The date palm sap from the individual clay pots typically contains 1–3 L of sap from a single tree; this sap is poured into a larger metal 22-L aluminum vessel with sap from several trees. Sellers will typically first walk to villages near where they collected the sap and either sell it door to door or from the road. If they still have some remaining, they will go to the market to sell it. Some buyers cook the sap to make molasses or special deserts; some is consumed fresh. Customers typically bring their own glass or jar, and the date palm sap seller dispenses the sap from the larger container. Fresh sap has to be sold early in the morning; otherwise, the sap will ferment and no longer taste sweet. The market price for fresh date palm sap was 8–10 taka (US $0.14–$0.17) per liter before 10:00 am and 4–5 taka (US $0.07–$0.09) after 10:00 am.

Owners of date palm trees reported that they often hear bats at night. Owners viewed the fruit bats as a nuisance because they frequently drink the palm sap directly from the tap or the clay pot. Bat excrement is commonly found on the outside of the clay pot or floating in the sap. Occasionally dead bats are found floating in the pots.

One of the Nipah case-patients who died was the son of a date palm sap collector. The collector harvested sap from his own date palm trees that grew in his household compound, as well from other trees in the area. The collector reported that he had recently heard bats near his trees at night and noted signs of bat excrement in and outside of the clay pot used to collect the sap. His son had been consuming date palm sap on a daily basis since the start of the season. Several days before the outbreak, the date palm sap collector sent a gift of fresh palm sap to his relatives living in a nearby homestead. Encephalitis developed in 3 members of the recipient household; 2 died.

## Discussion

This outbreak was almost certainly caused by infection with Nipah virus. The tight clustering of cases in time and space suggests a single etiologic agent. The clinical signs and symptoms of fever, central nervous system involvement, and rapid progression to death are consistent with other Nipah outbreaks in Bangladesh. The outbreak occurred in the same region and during the same time of year as the 4 prior confirmed Nipah outbreaks in Bangladesh. Finally, 2 of 3 persons who met the outbreak-associated encephalitis case definition and were able to provide a serum specimen had IgM antibodies against Nipah virus. The single outbreak-associated specimen that was IgM negative was drawn from a patient on day 2 after symptom onset, so IgM antibodies may not have been present in sufficient quantity to be detected (*20*).

Date palm sap was the likely vehicle of transmission for most of the Nipah virus infections in this outbreak. Drinking fresh, raw date palm sap was the sole exposure significantly associated with illness. Moreover, date palm sap is a biologically plausible vehicle. Although fruit bats uncommonly shed Nipah virus (*5**,**21*), when infected they can shed virus in both saliva and urine (*5**,**15**,**16*). Nipah virus can survive for days in fruit juice or flying-fox urine (*22*). Since date palm sap is sold and consumed within a few hours of collection, consumers could ingest infectious virus.

This outbreak provides further evidence that Nipah virus infection in humans in Bangladesh is a seasonal disease that results from interaction between P. giganteus fruit bats and humans. This is the fifth Nipah outbreak in 5 years that has been identified in the same region (Figure 1). A Nipah outbreak also was confirmed in Siliguri, India, 15 km north of the border with Bangladesh in January 2001 (*23*). Each of these outbreaks occurred between January and May. P. giganteus is widely distributed throughout Bangladesh (*24*). The reason the outbreaks are occurring in this region at this time of year may be related to a seasonal increase in Nipah virus shedding among P. giganteus or to P. giganteus' attraction to particular natural or agricultural foods that are seasonally available in this region and bring the bats into proximity with humans.

An important limitation of this investigation is that only 3 serum samples were available from case-patients. Thus, some persons included in the outbreak may not have had Nipah virus infection. Misclassifying non–case-patients as case-patients would bias the odds ratio toward null.

A second limitation is that proxy interviews were required to obtain exposure information for all case-patient interviews. In addition, the same population was used for in-depth interviews and for follow-up quantitative questionnaires. Thus, information bias is possible. However, proxy respondents were independently identified by the qualitative research team to ensure that only persons who directly observed the case-patients' exposures were included. The standardized questionnaire used for the case-control study was pretested. The interviewers were unaware of the study hypothesis, and respondents were encouraged to answer to the best of their knowledge. Discrepancies were found between the in-depth interviews and the quantitative studies. Three proxy respondents who unambiguously reported consuming date palm sap during the initial in-depth qualitative interview reported no consumption during the follow-up close-ended interview. These 3 persons were relatives of a date palm sap collector. Their answers to the case-control questionnaire were used in the analysis. Thus, some study participants probably did realize what some of the investigation team's hypotheses were; however, this factor apparently biased the data against finding an association with date palm sap. Indeed, none of these sources of bias were likely to produce a spurious association between disease and raw date palm sap consumption.

A third limitation is that Nipah virus was not isolated from date palm sap. Indeed, by the time the investigation implicated date palm sap, transmission of Nipah virus was no longer occurring in the area, and we did not collect date palm sap samples. However, the evidence favoring date palm juice as the vehicle for transmission of Nipah virus in this outbreak is stronger than for any alternative hypothesis.

This study highlights the value of a diverse outbreak investigation team. Clinicians identified and cared for ill patients. Experts in qualitative research collaborated with the epidemiology team to understand potential routes of exposure and then used in-depth discussion with affected community residents to identify locally relevant exposures and frame the questions for the quantitative investigation. By conversing with residents of the affected area, qualitative investigators corrected outsiders' misconceptions about local exposures and were also able to quickly develop locally relevant messages to prevent secondary transmission. Laboratory investigators confirmed the cause of the outbreak. A close working relationship between government health workers and researchers permitted shared access to relevant information that provided government authorities with information on how to manage the outbreak and prevent further transmission.

Investigation of different Nipah outbreaks in Bangladesh have identified different routes of transmission including climbing trees, contact with sick persons, and contact with sick animals (*9**–**13*). This investigation identifies another way that Nipah virus may be transmitted from P. giganteus to humans in Bangladesh. Fresh date palm sap is a national delicacy that is enjoyed by millions of Bangladeshis each winter. Apparently, most servings of fresh date palm sap are safe to drink. However, this investigation suggests that, at least occasionally, the sap contains a sufficient dose of Nipah virus to be fatal to humans. Further research to define how frequently this occurs is important. Persons who want to avoid ingesting Nipah virus from this route, should avoid drinking raw date palm sap. Low-cost interventions to restrict access of fruit bats to date palm taps and pots and that make fresh date palm sap safer should be developed and evaluated. In addition, continued research to better understand Nipah virus transmission between bats and the multiple pathways of human infection are important for developing sound prevention strategies.
